# Changes in prospectively collected patient-reported outcomes among women with incident endometrial cancer

**DOI:** 10.1007/s11764-024-01536-z

**Published:** 2024-01-24

**Authors:** Jennifer A. Sinnott, Elaheh Torkashvand, Caitlin E. Meade, Ritu Salani, Monica Hagan Vetter, Bobbie Hall, Rebecca Skolnick, Kristin L. Bixel, David E. Cohn, Casey M. Cosgrove, Larry J. Copeland, Courtney Hebert, Ashley S. Felix

**Affiliations:** 1 Department of Statistics, The Ohio State University College of Arts and Sciences, Columbus, OH USA; 2 Division of Epidemiology, The Ohio State University College of Public Health, 1841 Neil Avenue, 304 Cunz Hall, Columbus, OH 43210 USA; 3 Department of Obstetrics and Gynecology, University of California, Los Angeles, Los Angeles, CA USA; 4 Division of Gynecologic Oncology, Baptist Health Medicine Group, Lexington, KY USA; 5 Denison University, Granville, OH USA; 6 Division of Gynecologic Oncology, The Ohio State University College of Medicine, Columbus, OH USA; 7 Division of Biomedical Informatics, The Ohio State University College of Medicine, Columbus, OH USA

**Keywords:** Age, Anxiety, Depression, Lymphadenectomy, Minimally invasive surgery, Physical functioning, Trajectories

## Abstract

**Purpose:**

We examined associations between patient and treatment characteristics with longitudinally collected patient-reported outcome (PRO) measures to provide a data-informed description of the experiences of women undergoing treatment for endometrial cancer.

**Methods:**

We administered National Institutes of Health Patient Reported Outcomes Measurement Information System (PROMIS) questionnaires at the preoperative visit and at 6 and 12 months after surgery. Anxiety, depression, fatigue, sleep disturbance, pain, physical function, and ability to participate in social roles were assessed. Analysis of variance (ANOVA) and linear mixed models were used to examine associations between patient characteristics and PRO measures at baseline and through time.

**Results:**

Of 187 women enrolled, 174 (93%) and 103 (69%) completed the 6- and 12-month questionnaires, respectively. Anxiety was substantially elevated at baseline (half of one population-level standard deviation) and returned to general population mean levels at 6 and 12 months. Younger age, Medicaid/None/Self-pay insurance, prevalent diabetes, and current smoking were associated with higher symptom burden on multiple PRO measures across the three time points. Women with aggressive histology, higher disease stage, or those with adjuvant treatment had worse fatigue at 6 months, which normalized by 12 months.

**Conclusions:**

We observed a high symptom burden at endometrial cancer diagnosis, with most PRO measures returning to general population means by 1 year. Information on risk factor-PRO associations can be used during the clinical visit to inform supportive service referral.

**Implications for Cancer Survivors:**

These findings can inform clinicians’ discussions with endometrial cancer survivors regarding expected symptom trajectory following diagnosis and treatment.

**Supplementary Information:**

The online version contains supplementary material available at 10.1007/s11764-024-01536-z.

## Introduction

Although disease- and treatment-related symptoms such as pain, fatigue, and depression are common among cancer survivors, they are not always addressed in clinical care due to difficulties with in-person communication between providers and patients [1]. Recent pilot programs have sought to improve this situation by integrating patient-reported outcome (PRO) assessment through questionnaires administered prior to clinical appointments and during active follow-up [1–3]. Patients have expressed more positive healthcare experiences as a result, noting improved communication with providers and a greater sense of control of their care [3]. Preliminary work suggests these PRO programs could also improve medical care, as PRO measures are indicative of cancer progression and survival [4]. Some PRO measures, including fatigue or pain, may reflect comorbidities that could be ameliorated through referrals or other interventions. Recognizing the broad utility of these measures, the National Institutes of Health (NIH) developed the PRO Measurement Information System (PROMIS), which provides reliable, validated questionnaire-based tools for outcome assessment [5].

One cancer population that could experience an enormous benefit from PRO assessment is endometrial cancer survivors. Endometrial cancer is the most common gynecologic cancer in the USA with 66,200 new cases expected in 2023 [6]. Favorable prognosis along with increasing annual incidence has produced an estimated 891,560 endometrial cancer survivors, making this the second largest population of female cancer survivors in the USA [7]. As these women typically have many years of life after diagnosis and treatment, a better understanding of their lived experience could have a significant impact on quality of life (QOL). In a systematic review of 27 published manuscripts examining PRO measures among women with endometrial cancer, our group reported that most PRO studies implement questionnaires several years following endometrial cancer diagnosis and primary treatment [8]. This represents a missed opportunity to understand the symptom burden early in the care trajectory. Baseline information would allow clinicians to refer women to appropriate services to ameliorate symptoms and establish a reference point to quantify changes stemming from treatment or supportive care interventions. In addition, understanding which women are more likely to experience certain symptoms could provide opportunities for clinicians to proactively engage with high-risk patients. Therefore, we implemented NIH PROMIS questionnaires to women with a preoperative diagnosis of endometrial cancer and followed women for 1 year after their diagnosis to determine symptom magnitude and relevant predictors.

## Methods

### Study population

We conducted a prospective cohort study of women scheduled to undergo a hysterectomy for primary treatment of endometrial cancer. Women were included if they were at least 18 years of age, had no previous cancer diagnosis (excluding skin cancer), spoke English, were not cognitively impaired [assessed by pre-screening patient EHRs for cognitive impairment diagnoses (e.g., dementia) or reports from the nurse/provider to not approach due to cognitive impairment], and were able to provide informed consent. Women who only had follow-up care at our institution or who had chemotherapy or radiation therapy prior to the surgery were excluded. Trained research staff conducted eligibility screening, obtained informed consent, and enrolled women during their preoperative visit; women could select either a short form on paper or iPad, or a patient portal–based questionnaire, as detailed below. Enrollment began in November 2017 and concluded in March 2021. Women completed baseline PRO questionnaires in clinic prior to hysterectomy and at the 6- and 12-month follow-up appointments. If women were unable to return to clinic, research staff collected PRO responses by telephone or mail (short-form participants only). The 12-month questionnaire was added after study initiation; as such, only the women who were still within the 12-month time frame for participation were asked to complete the 12-month questionnaire. The study was approved by the OSU Institutional Review Board and all participants provided written informed consent.

### Patient-reported outcomes assessment

Participants completed the NIH PROMIS questionnaires, which are a set of valid and reliable measures used to assess symptoms, function, and various aspects of health-related quality of life [9]. Participants were given the option of completing the *PROMIS-29 Profile v2.0* and the *PROMIS Sexual Function Profile v1.0—Female*, two brief, fixed-length paper forms, or a questionnaire using computer-adaptive testing (CAT) implemented in the Epic (Epic Systems, Verona WI) electronic health record (EHR) through a patient-facing portal (MyChart). While the *PROMIS-29* short forms ask a standard set of questions across participants, the CAT tool uses item response theory (IRT) to reduce the number of items used to measure specific symptoms and health status domains. IRT allows for improvement of items and assembles domains of items which are unidimensional and not excessively redundant. The short-form scores are intended to produce scores that would match those obtained from CAT, although measurement error is slightly greater with short forms [10].

This analysis focused on the domains of anxiety, depression, fatigue, sleep disturbance, pain interference, global pain intensity (short form only), physical function, and ability to participate in social roles. Few women (*n* = 18) completed the Sexual Function Profile; thus, we omit those results here. Raw PRO scores were normalized to a mean score of 50 with standard deviation of 10 (where 50 represents the mean of the general population) using the HealthMeasures scoring system. High scores represent more of the domain being measured. On symptom-oriented domains (anxiety, depression, fatigue, sleep disturbance, and pain interference), higher scores represent worse symptoms. On function-oriented domains (physical function and ability to participate in social roles), higher scores represent better functioning. At the time the PRO instruments were implemented into our EPIC system, the one-item global pain intensity question was not available. Therefore, women who completed the CAT PROMIS questionnaire are missing data for this domain. Raw scores for global pain intensity range from 0 to 10 (0 = No Pain to 10 = Worst Imaginable Pain) [11].

### Electronic health record data

Information from the EHR, including age at diagnosis (< 50, 50–64, ≥ 65), self-reported race (White, Black, Other; collapsed into White, Non-White), insurance status (private, Medicare, Medicaid, Military/Tricare, none/self-pay; collapsed into Medicare/Military/Tricare, Medicaid/None/Self-pay, and private), hypertension (no vs. yes), diabetes (no vs. yes), history of alcohol use (no vs. yes), smoking status (never smoker, former smoker, current smoker), and distance from treatment facility (< 50 miles, 50–99 miles, ≥ 100 miles), was collected. We abstracted height and weight measured at the preoperative visit to calculate body mass index (BMI) and categorized women as defined by the World Health Organization (WHO): (non-Asian women) normal weight 18.5–24.9 kg/m^2^, overweight 25–29.9 kg/m^2^, and obese ≥ 30 kg/m^2^; (Asian women) normal weight 18.5–22.9 kg/m^2^, overweight 2–24.9 kg/m^2^, and obese ≥ 25 kg/m^2^. Information on the following tumor and surgical characteristics was available from the pathology and operative reports: surgical approach (robotic-assisted laparoscopy, laparotomy), lymph node staging (not performed, sentinel ± para-aortic lymphadenectomy, pelvic ± para-aortic lymphadenectomy), histology (low-grade endometrioid, high-grade endometrioid, serous, carcinosarcoma, clear cell, mixed epithelial, dedifferentiated/undifferentiated endometrial; collapsed into low-grade endometrioid vs. high-grade endometrioid/non-endometrioid), and stage (I, II, III, IV; collapsed into I vs. II/III/IV). Information on adjuvant treatment included chemotherapy received (no vs. yes) and radiation received (no vs. yes).

### Statistical analysis

Baseline and clinical characteristics of the overall study population were summarized with counts and percentages. We calculated the means and standard deviations of each PRO at baseline within levels of baseline variables (age, race, insurance, BMI, hypertension, diabetes, alcohol use, smoking status, distance from treatment facility, and questionnaire type [short form vs. CAT]) and assessed differences using a one-way analysis of variance (ANOVA) *p*-value. We tested for differences between mean PRO measures in our study population and the general population (50) using *t*-tests at each time point. To evaluate whether PRO measures varied over time, we used linear mixed effects regression models with the PRO as the outcome and time as the only (fixed effect) covariate, with a random intercept for individual to account for the repeated measures. All women with the PRO measured at least once were included in the model.

Next, to examine how patient characteristics were associated with how PRO measures changed through time, we used linear mixed effects models with each patient characteristic, time, and a multiplicative interaction between the characteristic and time as fixed effects, and a random intercept for individual. We visualized these relationships by plotting PRO means through time within levels of each covariate. As a sensitivity analysis, we compared baseline characteristics and baseline PRO scores between women who participated in the 12-month survey (*n* = 103) and women who were eligible to participate yet declined (*n* = 47). Statistical analyses were performed in SAS version 9.4 or R version 4.2.2, using R packages lme4 [12] and lmerTest [13]. All *p*-values were two-sided; *p*-values less than 0.05 were considered significant.

## Results

### Study population

We approached 352 potentially eligible participants, and of these, 226 (64.2%) consented to participate. Of the 226 consented, 11 women were ineligible based on inclusion criteria, 27 did not complete a baseline questionnaire, and 1 woman was excluded for missing baseline PROMIS *T*-scores due to a computer error, leaving 187 in the analytic sample. Questionnaire completion was 93% at 6 months (*n* = 174) and 68.7% at 12 months (*n* = 103). Because the 12-month questionnaire was added after study initiation, it was only requested from 150 women.

Baseline characteristics of women who participated in the study are shown in Table 1. Most women were White (95.7%) and obese (75.4%); median age at diagnosis was 62 years. Just over half (52.5%) of women lived ≥ 50 miles from the hospital, and just under half (46.5%) had private insurance. Most of the cohort were never smokers (65.8%), and 44.4% reported alcohol use. Hypertension (54.6%) and diabetes (21.4%) were common. Most (93.1%) had robotic-assisted laparoscopic surgery and 64.2% had sentinel ± para-aortic lymphadenectomy. Low-grade endometrioid (78.1%) and stage I disease (78.6%) were common. Approximately one-quarter of the cohort received chemotherapy (24.6%) and/or radiation (24.6%).

**Table 1:** Baseline patient demographic and clinical characteristics of the overall study population

	Overall (*n* = 187)
Characteristics	*n* (%)
Age	
< 50	20 (10.7)
50–64	94 (50.3)
≥ 65	73 (39.0)
Median (IQR)	62 (55–69)
Race	
White	179 (95.7)
Black	5 (2.7)
Other	3 (1.6)
Insurance status	
Medicare	77 (41.2)
Medicaid	21 (11.2)
Private	87 (46.5)
Military/Tricare	1 (0.5)
None/Self-pay	1 (0.5)
BMI^1^	
Normal weight	11 (5.9)
Overweight	35 (18.7)
Obese	141 (75.4)
Hypertension	
No	85 (45.5)
Yes	102 (54.6)
Diabetes	
No	147 (78.6)
Yes	40 (21.4)
Alcohol use	
No	103 (55.1)
Yes	83 (44.4)
Unknown	1 (0.5)
Smoking status	
Never smoker	123 (65.8)
Former smoker	43 (23.0)
Current smoker	21 (11.2)
Distance from treatment facility	
< 50 miles	89 (47.6)
50–99 miles	79 (42.3)
≥ 100 miles	19 (10.2)
Questionnaire type	
Computer-adaptive testing	87 (46.5)
Short form	100 (53.5)
Histology	
Low-grade endometrioid	146 (78.1)
High-grade endometrioid	7 (3.7)
Serous	15 (8.0)
Carcinosarcoma	3 (1.6)
Clear cell	5 (2.7)
Mixed cell	6 (3.2)
Dedifferentiated/undifferentiated	5 (2.7)
Stage	
I	147 (78.6)
II	4 (2.1)
III	31 (16.6)
IV	5 (2.7)
Surgical approach	
Robotic-assisted laparoscopy	174 (93.1)
Laparotomy	14 (7.5)
Lymph node staging	
Not performed	14 (7.5)
Sentinel ± para-aortic lymphadenectomy	120 (64.2)
Pelvic ± para-aortic lymphadenectomy	53 (28.3)
Chemotherapy	
No	139 (74.3)
Yes	46 (24.6)
Unknown	2 (1.1)
Radiation	
No	139 (74.3)
Yes	46 (24.6)
Unknown	2 (1.1)

^1^The World Health Organization (WHO) categorizes Underweight/Normal weight for White, Black, and Hispanic individuals as < 25.0 kg/m^2^, Overweight as 25 to 29.9 kg/m^2^, Class I Obesity as 30 to 34.9 kg/m^2^, Class II Obesity as 35 to 39.9 kg/m^2^, and Class III Obesity as greater than or equal to 40.0 kg/m^2^. For Asian and South Asian populations, Underweight/Normal weight is a BMI classified as < 23.0 kg/m^2^, Overweight as 23 to 24.9 kg/m^2^, and Obese as ≥ 25.0 kg/m^2^

All variables (except questionnaire type) were abstracted from the EHR

### Baseline characteristics and baseline PROMIS *T*-scores

Associations between baseline characteristics and baseline PROMIS *T*-scores are shown in Table 2. Significant differences by age were observed for baseline anxiety (*p* < 0.001), depression (*p* < 0.001), fatigue (*p* < 0.001), sleep disturbance (*p* < 0.001), and pain interference (*p* = 0.009). Generally, younger vs. older women reported higher anxiety, depression, fatigue, sleep disturbance, and pain interference. Non-White women experienced higher pain interference than White women (*p* = 0.04). There were some differences with respect to insurance status for anxiety (*p* = 0.006), depression (*p* < 0.001), fatigue (*p* = 0.008), sleep disturbance (*p* = 0.001), pain interference (*p* = 0.003), and physical function (*p* = 0.03). Women with Medicaid/None/Self-pay generally reported worse levels of these PROMIS *T*-scores than women with Medicare/Military/Tricare. Women with private insurance usually had PRO levels worse than women in the Medicare/Military/Tricare but better than Medicaid/None/Self-pay groups. There were differences by BMI in fatigue (*p* < 0.001), pain interference (*p* < 0.001), global pain (*p* = 0.02), physical function (*p* < 0.001), and social roles (*p* = 0.001). Women with higher BMI reported higher fatigue, pain interference, and global pain intensity, along with lower physical function and ability to participate in social roles. Women with hypertension reported worse physical function (*p* = 0.001), but no other differences compared to women without hypertension, while women with diabetes reported higher depression (*p* = 0.007), fatigue (*p* = 0.005), pain interference (*p* < 0.001), and global pain (*p* = 0.04), and less physical function (*p* = 0.002) and ability to participate in social roles (*p* = 0.002) than women without diabetes. There were differences by smoking status for anxiety (*p* = 0.03), fatigue (*p* = 0.04), sleep disturbance (*p* = 0.007), pain interferences (*p* = 0.02), and physical function (*p* = 0.04). Current smokers reported more anxiety, fatigue, sleep disturbance, pain interference, and lower physical function compared to never smokers; the values for former smokers were sometimes closer to those of current smokers and sometimes closer to those of never smokers depending on the PRO. There were no associations between PRO measures and alcohol use or distance from the treatment facility. Apart from physical function (*p* = 0.04), baseline PRO measures did not differ by assessment tool (short form vs. CAT).

**Table 2:** Means and standard deviations of baseline patient-reported outcome *T*-scores according to baseline characteristics

	Anxiety		Depression		Fatigue		Sleep disturbance		Pain interference		Global pain		Physical function		Social roles	
	(*n* = 187)		(*n* = 187)		(*n* = 187)		(*n* = 187)		(*n* = 186)		(*n* = 100)		(*n* = 187)		(*n* = 186)	
Characteristics	Mean (SD)	*p*^2^	Mean (SD)	*p*^2^	Mean (SD)	*p*^2^	Mean (SD)	*p*^2^	Mean (SD)	*p*^2^	Mean (SD)	*p*^2^	Mean (SD)	*p*^2^	Mean (SD)	*p*^2^
Age		< 0.001		0.001		< 0.001		< 0.001		0.009		0.08		0.85		0.17
< 50	62.3 (10.5)		55.4 (8.4)		59.1 (10.7)		57.9 (8.0)		57.0 (10.9)		4.6 (1.8)		46.7 (8.3)		49.9 (8.1)	
50–64	57.4 (8.6)		51.9 (7.9)		51.9 (10.3)		53.1 (9.2)		52.3 (10.5)		3.4 (2.8)		47.6 (8.7)		53.1 (9.1)	
≥ 65	54.1 (7.8)		48.8 (7.0)		48.9 (9.4)		48.4 (8.8)		49.5 (9.0)		2.6 (2.6)		46.9 (8.2)		54.1 (8.3)	
Race		0.99		0.99		0.49		0.06		0.04		0.12		0.13		0.41
White	56.6 (8.9)		51.0 (7.9)		51.4 (10.5)		51.5 (9.3)		51.4 (10.1)		3.1 (2.6)		47.4 (8.3)		53.3 (8.5)	
Non-White	56.7 (9.3)		51.1 (7.4)		54.0 (7.4)		57.9 (8.3)		58.9 (8.9)		5.2 (3.6)		42.8 (10.7)		50.6 (13.6)	
Insurance status		0.006		< 0.001		0.008		0.001		0.003		0.05		0.03		0.29
Medicare/Military/Tricare	54.5 (7.8)		49.1 (7.1)		49.3 (9.7)		48.8 (8.9)		49.8 (9.5)		2.6 (2.7)		46.8 (8.2)		53.9 (8.6)	
Medicaid/None/Self-pay	60.6 (10.6)		56.6 (9.8)		56.7 (11.3)		55.0 (10.9)		58.0 (11.1)		4.6 (2.6)		43.4 (9.3)		50.5 (11.0)	
Private	57.6 (8.9)		51.4 (7.3)		52.1 (10.2)		53.6 (8.7)		51.8 (9.9)		3.3 (2.5)		48.6 (8.1)		53.2 (8.2)	
BMI^1^		0.85		0.21		< 0.001		0.99		< 0.001		0.02		< 0.001		0.001
Normal weight	55.3 (10.9)		48.8 (7.0)		41.2 (7.4)		51.4 (8.7)		44.9 (8.5)		1.7 (2.0)		54.3 (7.0)		60.2 (5.8)	
Overweight	56.5 (9.4)		49.4 (6.9)		48.3 (7.9)		51.8 (7.5)		46.7 (8.3)		2.0 (1.9)		51.1 (6.9)		56.3 (6.4)	
Obese	56.8 (8.6)		51.6 (8.1)		53.1 (10.5)		51.8 (9.9)		53.5 (10.1)		3.7 (2.8)		45.7 (8.3)		51.8 (9.0)	
Hypertension		0.89		0.24		0.92		0.30		0.26		0.20		0.001		0.15
No	56.5 (9.0)		50.3 (7.8)		51.6 (10.3)		52.6 (9.4)		50.8 (10.1)		2.8 (2.3)		49.5 (8.1)		54.2 (8.2)	
Yes	56.7 (8.8)		51.6 (7.9)		51.4 (10.5)		51.1 (9.4)		52.5 (10.2)		3.5 (2.9)		45.3 (8.2)		52.3 (9.1)	
Diabetes		0.29		0.007		0.005		0.08		< 0.001		0.04		0.002		0.002
No	56.3 (8.5)		50.2 (7.8)		50.4 (10.2)		51.1 (8.9)		50.3 (9.9)		2.9 (2.7)		48.2 (8.3)		54.2 (8.4)	
Yes	58.0 (9.9)		54.0 (7.4)		55.6 (10.2)		54.1 (10.8)		57.0 (9.5)		4.2 (2.2)		43.6 (7.8)		49.4 (8.9)	
Alcohol use		0.88		0.83		0.97		0.55		0.45		0.70		0.37		0.93
No	56.4 (8.9)		51.0 (8.3)		51.6 (11.1)		51.2 (10.2)		52.5 (11.0)		3.4 (3.0)		46.7 (8.8)		53.0 (9.4)	
Yes	57.0 (8.9)		51.0 (7.3)		51.4 (9.5)		52.4 (8.3)		50.7 (9.0)		3.0 (2.0)		47.8 (7.9)		53.4 (8.0)	
Unknown	57.5 (NA)		55.9 (NA)		53.1 (NA)		58.7 (NA)		55.7 (NA)		2.0 (NA)		56.9 (NA)		51.8 (NA)	
Smoking status		0.03		0.53		0.04		0.007		0.02		0.08		0.04		0.18
Never smoker	56.6 (8.4)		50.6 (7.5)		50.5 (10.0)		51.2 (8.8)		50.3 (9.4)		2.8 (2.4)		48.4 (8.4)		54.0 (8.0)	
Former smoker	54.6 (9.2)		51.4 (7.9)		52.1 (10.3)		50.6 (10.1)		53.7 (10.7)		4.2 (2.8)		45.0 (8.6)		51.5 (10.7)	
Current smoker	61.0 (9.6)		52.7 (9.9)		56.5 (11.6)		57.8 (9.6)		55.7 (12.1)		3.1 (3.5)		45.2 (7.1)		51.5 (8.1)	
Distance from treatment facility		0.32		0.15		0.52		0.56		0.48		0.92		0.87		0.48
< 50 miles	57.6 (9.0)		51.8 (7.4)		51.8 (10.5)		52.4 (9.0)		51.2 (10.4)		3.2 (2.7)		47.5 (8.7)		52.4 (8.9)	
50–99 miles	55.9 (8.6)		50.9 (8.4)		51.8 (10.4)		51.5 (9.6)		52.7 (10.2)		3.3 (2.8)		46.9 (8.3)		53.6 (8.7)	
≥ 100 miles	55.1 (8.9)		47.9 (6.9)		48.9 (9.3)		50.0 (10.4)		50.0 (8.7)		2.9 (2.2)		47.6 (7.5)		54.7 (8.5)	
Questionnaire type		0.35		0.56		0.58		0.52		0.25				0.04		0.52
Computer-adaptive testing	57.3 (7.9)		51.4 (7.3)		52.0 (9.3)		52.3 (9.5)		50.8 (10.2)		–		45.9 (7.5)		53.6 (8.3)	
Short form	56.1 (9.6)		50.7 (8.3)		51.1 (11.2)		51.4 (9.3)		52.5 (10.1)		–		48.4 (9.0)		52.8 (9.2)	

^1^The World Health Organization (WHO) categorizes Underweight/Normal weight for White, Black, and Hispanic individuals as < 25.0 kg/m^2^, Overweight as 25 to 29.9 kg/m^2^, Class I Obesity as 30 to 34.9 kg/m^2^, Class II Obesity as 35 to 39.9 kg/m^2^, and Class III Obesity as greater than or equal to 40.0 kg/m^2^. For Asian and South Asian populations, Underweight/Normal weight is a BMI classified as < 23.0 kg/m^2^, Overweight as 23 to 24.9 kg/m^2^, and Obese as ≥ 25.0 kg/m^2^

^2^*p*-values are from an overall ANOVA *F*-test

### PROMIS *T*-score trajectories

Table 3 shows PROMIS *T*-scores for PRO measures (global pain intensity scores range from 0 to 10) at each of the three time points. At baseline, anxiety, fatigue, sleep disturbance, pain interference, and physical function were significantly worse than the general population. In particular, anxiety was elevated by over half of the population-level standard deviation. Only ability to participate in social roles was significantly better in our study population compared with the general population (53.2 vs. 50.0). Reports of anxiety (*p* < 0.001), depression (*p* = 0.04), sleep disturbance (*p* = 0.03), and global pain intensity (*p* = 0.004) varied significantly through time, with average scores for these domains being highest before surgery and lower by the 6- and 12-month post-surgery time points. Other PRO measures did not significantly vary over the 1-year follow-up in these unadjusted analyses.

**Table 3:** Means and standard deviations of patient-reported outcome T-scores at three time points

PROMIS domain	Baseline			6-month			12-month			
	*N*	Mean (SD)	*p*^1^	*N*	Mean (SD)	*p*^1^	*N*	Mean (SD)	*p*^1^	*p*^2^
Anxiety	187	56.6 (8.9)	< 0.001	172	51.1 (9.5)	0.14	103	51.2 (9.3)	0.20	< 0.001
Depression	187	51.0 (7.9)	0.07	173	49.8 (8.8)	0.81	102	49.3 (9.9)	0.47	0.04
Fatigue	187	51.5 (10.4)	0.05	173	51.3 (10.2)	0.09	103	49.1 (9.7)	0.33	0.20
Sleep disturbance	187	51.8 (9.4)	0.01	173	50.4 (8.8)	0.51	103	49.3 (9.1)	0.46	0.03
Pain interference	186	51.7 (10.2)	0.02	173	50.8 (9.3)	0.25	103	49.8 (9.3)	0.85	0.61
Global pain^3^	100	3.2 (2.7)	-	93	2.5 (2.6)	-	44	2.1 (2.6)	-	0.004
Physical function	187	47.2 (8.4)	< 0.001	174	47.2 (8.7)	< 0.001	103	47.6 (8.7)	0.007	0.87
Social roles	186	53.2 (8.7)	< 0.001	172	52.6 (10.1)	0.001	103	54.8 (9.4)	< 0.001	0.24

^1^*p*-value from one-sample *t*-test comparing mean in the study population at specific time point to the general population (mean = 50, standard deviation = 10)

^2^*p*-value from linear mixed effects model for difference in means across time points

^3^Global pain scale 0–10

### Relationships between covariates and PRO measures through time

**Fig. 1:**
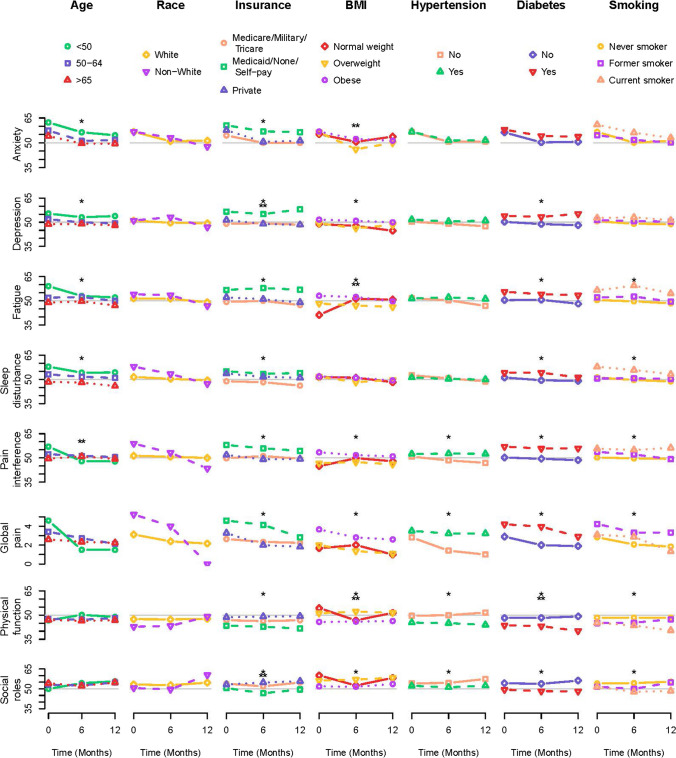
For each patient-reported outcome *T*-score, mean values through time are plotted by levels of each demographic and lifestyle covariate. Values are plotted at baseline (0 months), 6 months, and 12 months, with lines connecting the points through time. A single star indicates that the main effect of the covariate was statistically significant in a linear mixed effects model including the covariate, time, and the interaction between the covariate and time. A significant main effect means that the mean values of the *T*-score differ by levels of the categorical variable regardless of time. A double star indicates that the interaction was statistically significant. A significant interaction means that the relationship between the mean values of the *T*-score and the categorical variable changes through time

**Fig. 2:**
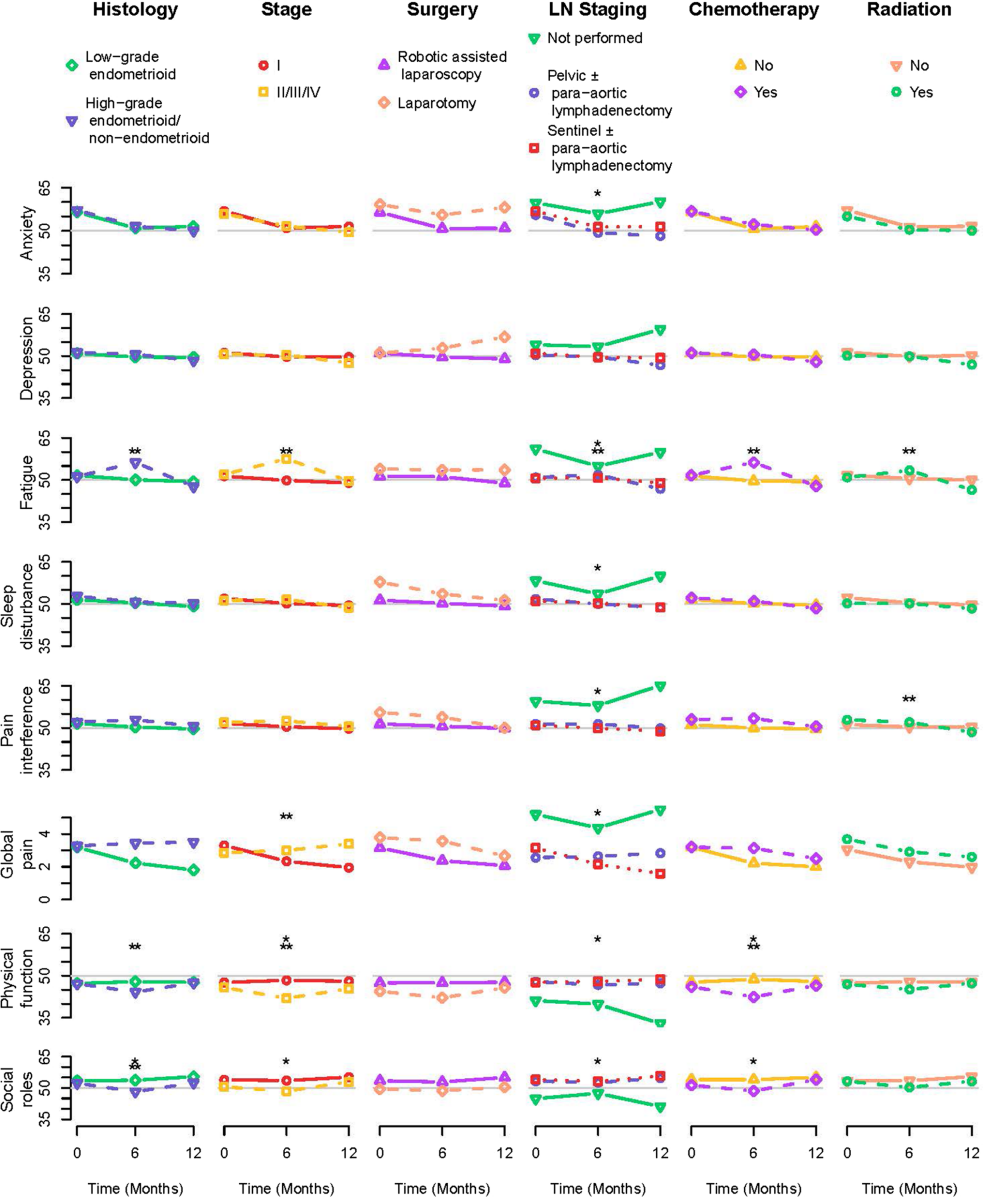
For each patient-reported outcome *T*-score, mean values through time are plotted by levels of each tumor and treatment covariate. Values are plotted at baseline (0 months), 6 months, and 12 months, with lines connecting the points through time. A single star indicates that the main effect of the covariate was statistically significant in a linear mixed effects model including the covariate, time, and the interaction between the covariate and time. A double star indicates that the interaction was statistically significant

Figures 1 and 2 display mean PRO values by levels of demographic, lifestyle, tumor, and treatment characteristics through time, and statistical significance of the main effects and interactions is indicated. The means plotted here (and standard deviations and *p*-values) are presented in Supplementary Table 1. Alcohol use, distance to treatment facility, and questionnaire type are not presented in Figs. 1 and 2 due to lack of significance; however, the associations are available in the supplementary table.

#### Anxiety

Younger women experienced higher anxiety throughout follow-up, though for all women anxiety is highest before surgery at baseline. Women in the Medicaid/self-payer/no insurance group also reported more anxiety through follow-up. Obese women experienced the highest anxiety at baseline which declined over time; normal and overweight women, on the other hand, experienced a drop in anxiety at 6 months and an increase at 12 months. Women who did not have lymph node staging had heightened anxiety throughout the time period, and those with sentinel lymphadenectomy with or without para-aortic lymphadenectomy had slightly higher anxiety on average than those with pelvic lymphadenectomy with or without para-aortic lymphadenectomy.

#### Depression

Depression was higher through time among younger women, women with Medicaid insurance, no insurance, or self-payers, obese women, and women with diabetes.

#### Fatigue

Fatigue was highest at baseline among younger women, but age-related differences attenuated over time. Fatigue was consistently higher among women in the Medicaid/None/Self-Pay group, those with diabetes, and current smokers. Among overweight and obese women, fatigue declined during the 1-year follow-up, while among normal weight women fatigue was low at baseline and increased at 6 and 12 months. Fatigue peaked at 6 months for women with high-grade endometrioid or non-endometrioid histology, higher stage, those treated with chemotherapy, and those treated with radiation. Women with no lymph node staging experienced high fatigue particularly at baseline and 12 months.

#### Sleep disturbance

Younger women experienced more sleep disturbance, though declines over time were observed for women of all ages. Sleep disturbance was higher among women with Medicaid, no insurance, self-pay, and private insurance through time, higher among women with diabetes, and higher among current smokers. Sleep disturbance was significantly elevated at baseline among women who did not receive lymph node staging, with a decline at 6 months followed by an increase at 12 months.

#### Pain interference and global pain

For the most part, risk factor associations for pain interference and global pain were similar. Medicaid/None/Self-pay insurance, obesity, hypertension, diabetes, and absence of lymph node staging were related to higher levels of pain interference and global pain. Smoking status showed slightly different associations for these two PRO measures, with current smokers experiencing the highest levels of pain interference and former smokers experiencing the highest levels of global pain. Women with stage I disease experienced higher global pain at baseline which diminished, while women with stages II–IV disease had increasing global pain through time. Age was only associated with pain interference, with younger women experiencing high levels of pain interference at baseline, which subsequently declined over time. Finally, women treated with radiation had higher pain interference at baseline, which declined over time.

#### Physical function

Women with Medicaid/None/Self-pay, hypertension, diabetes, and either current or former smoking had lower physical function, as did women with no lymph node staging. Normal weight women experienced a decline in physical function at 6 months and a return by 12 months. Similar patterns were observed among women with high-grade endometrioid/non-endometrioid histology, higher stage, and those treated with chemotherapy.

#### Social roles

Women with Medicaid/None/Self-Pay insurance, obesity, hypertension, diabetes, current or former smoking, higher stage cancer, chemotherapy, and no lymph node staging experienced worse ability to participate in social roles. Women with high-grade endometrioid/non-endometrioid histology reported worse ability to participate in social roles at 6 months, which improved by 12 months.

In a sensitivity analysis comparing baseline characteristics and mean PRO scores between women who did (*n* = 103) and did not (*n* = 47) participate in the 12-month survey, we observed that women who did not participate were more commonly current vs. never smokers and baseline PRO scores for fatigue, sleep disturbance, pain interference, and participation in social roles were worse in this group (data not tabled).

## Discussion

In this single-institution study of women newly diagnosed with endometrial cancer, we used an easily implementable tool to characterize the preoperative symptom burden and longitudinal changes in symptoms. At baseline, self-reports of anxiety, fatigue, sleep disturbance, pain interference, and physical function were significantly worse than the general population; however, most symptoms improved by the 6-month follow-up except for physical function, which remained significantly worse than the general population over the 12-month follow-up. We also noted important risk factor-symptom associations, some of which have not been previously reported, potentially directing clinicians to focus clinical care discussions on specific patient-related factors that impact symptom trajectory.

Few studies have examined longitudinal patterns of the patient-reported symptom burden among women with endometrial cancer. In a systematic review, we observed that PRO studies of women with endometrial cancer were mostly cross-sectional with PRO assessment occurring at some point in time after the endometrial cancer diagnosis [8]. While these studies are important for providing a snapshot of the endometrial cancer symptom burden, they limit opportunities for clinicians and patients to proactively discuss and manage symptoms early in the treatment process and potentially introduce survivorship bias. Moreover, these studies were limited by a one-time assessment of PRO measures, preventing an understanding of the dynamic picture of symptoms that evolves over the survivorship period. Another distinction of the current study is the exclusive focus on women with endometrial cancer. In prior longitudinal PRO studies, women with endometrial cancer have been included with other gynecologic cancer survivors [14]; however, women with endometrial cancer may have a unique experience that is masked in these analyses.

As observed in the present study and by other studies of women newly diagnosed with endometrial cancer [15, 16], the most impaired area at baseline was anxiety, with mean level at the approximate 75th percentile of the population at baseline returning close to the general population mean (50th percentile) a few months after diagnosis. This finding reinforces the need to provide reassurance to endometrial cancer survivors at the time of diagnosis and throughout the treatment process. Further, while anxiety can be considered a normal reaction to an endometrial cancer diagnosis, our data suggests that sustained anxiety after the 6-month mark is not part of the typical experience. As such, continued reports of anxiety should be met with additional conversations and potential referral to supportive services.

Cross-sectional associations between patient characteristics and PRO measures among endometrial cancer survivors have mostly focused on the role of BMI [8]. In line with prior studies, we observed that obese women reported lower physical function; however, we additionally observed obesity to be related to higher fatigue, pain interference, and global pain intensity and lower ability to participate in social roles at baseline. Our analyses incorporating follow-up time suggest that anxiety declined among obese women while normal weight and overweight women experienced increased anxiety at the 1-year mark. In addition, over time, fatigue and physical function improved among obese women, while normal weight women experienced worse fatigue and physical function at 6 and 12 months. These findings might reflect that normal weight women are more likely to develop high-grade endometrioid and non-endometrioid tumors, which carry a higher likelihood of receiving adjuvant treatment. In line with this notion, we also observed worse fatigue associated with chemotherapy and radiation, which peaked 6 months after surgery.

In addition, we observed that older women were significantly less likely to report anxiety, depression, sleep disturbance, and fatigue compared with women diagnosed with endometrial cancer at younger ages. We suspect that younger women with a cancer diagnosis may be balancing other demands that lead to these symptoms being more impactful in their cancer journey. As such, younger endometrial cancer survivors might require unique resources to support them during their cancer journey. Current smokers and those with diabetes reported worse pain interference and sleep disturbance compared with never smokers and non-diabetics, respectively. In addition, women reporting current smoking had higher fatigue and worse physical functioning, while women with diabetes were more likely to report depressive symptoms and have lower ability to participate in social roles. These associations provide another tool that clinicians might use to motivate behavior change (i.e., smoking cessation), in addition to aligning with cardiovascular health recommendations. Our study population included few non-White women with endometrial cancer; however, baseline pain interference and sleep disturbance were borderline worse in non-White as compared with White patients. These findings should be evaluated in cohorts with better representation of racially diverse patients.

The recent incorporation of PRO assessments into randomized clinical trials of endometrial cancer patients provides opportunities to assess the role that treatment plays on PRO measures [17]. These analyses are particularly relevant when no superior treatment is identified; without a clear survival advantage, information regarding the symptom burden associated with treatment could aid the decision-making process. As noted, our analyses suggest that treatment with adjuvant radiation or chemotherapy resulted in higher fatigue, while adjuvant radiation was associated with worse pain interference, and adjuvant chemotherapy was related to worse physical functioning. The finding of worse physical functioning associated with chemotherapy concurs with a recent analysis of 287 endometrial cancer survivors in the Women’s Health Initiative Life and Longevity after Cancer observational study [18]. Our results related to treatment effects on PRO measures are not directly comparable to reports from Gynecologic Oncology Group (GOG)-258, given the enrollment of women with advanced endometrial cancer and the comparison of new treatments with standard of care (e.g., all women received adjuvant treatment). In a secondary analysis of GOG-258 patients, chemotherapy plus radiation was related to worse health-related quality of life and greater gastrointestinal toxicity compared to women receiving radiation only [17]. Our findings agree with a recent secondary data analysis of the Netherlands Biomarker Guided Treatment in Gynaecological Cancer Trial (MoMaTEC2), where adjuvant chemotherapy was associated with reduced functioning and a higher symptom burden [19]. However, in MoMaTEC2, surgical staging was unrelated to quality of life or symptoms, whereas we observed lower pain intensity and lower pain interference among women who had either pelvic or sentinel lymphadenectomy (with or without para-aortic lymph node sampling) compared with women who did not have lymph node staging. While surprising, this may be related to administration of pain medications for women undergoing these procedures, which might result in a better pain-related profile. Alternatively, morbidly obese women are most likely to not have lymph node staging, and in our study population, obesity was significantly associated with worse global pain and pain interference.

The longitudinal evaluation of PRO measures in endometrial cancer survivors is important for several reasons. First, these assessments provide an opportunity to understand the unique survivorship experience of women with endometrial cancer for the optimization of clinical care and provision of supportive services. Moreover, understanding relationships between modifiable factors and PRO measures represents an opportunity to manage symptoms through health behavior change. Finally, PRO assessments might serve as important conversation starters between patients and clinicians and empower patients to participate more in their treatment and survivorship planning.

Limitations of our study include the small sample size, lack of racial and ethnic diversity (reflective of the geographical location of the institution), focus on individual-level determinants of PRO measures, potential selection bias, and sample attrition. While an understanding of relationships between patient characteristics and PRO measures is an important first step in guiding patient-provider discussions of symptoms, an understanding of how multilevel determinants influence PRO measures would provide greater context. The potential for selection and attrition biases to influence our results is important, particularly if women with better symptoms were more likely to agree to participate and respond to the follow-up questionnaires than those with worse symptoms. However, strengths of this analysis include enrollment of women during the preoperative visit, longitudinal assessment of PRO measures, and abstraction of electronic health record data.

In conclusion, we identified a clinically significant symptom burden at the preoperative endometrial cancer visit, which generally improved with greater time since the diagnosis. Future studies focused on the trajectory of symptoms following primary endometrial cancer treatment and survival outcomes will be conducted in this cohort to advance our understanding of prognostic significance of PRO measures in this patient population.

## Supplementary Information

Below is the link to the electronic supplementary material.

Supplementary file1 (PDF 59 KB)

## Data Availability

Requests to access the data set from qualified researchers may be sent to the corresponding author.
