# Supplementary material for: Changes in prospectively collected patient-reported outcomes among women with incident endometrial cancer
Source: J Cancer Surviv. 2024 Jan 24;19(4):1119–31. doi: 10.1007/s11764-024-01536-z (PMC12283808; doi:10.1007/s11764-024-01536-z)
Supplement: Supplementary file 1 — Supplementary file1 (PDF 59 KB) [file 11764_2024_1536_MOESM1_ESM.pdf]

Supplementary Table 1. Means and standard deviations of patient-reported outcomes at three time points by levels of covariates

	Anxiety					Depression					Fatigue				
	Baseline	6 Months	12 Months			Baseline	6 Months	12 Months			Baseline	6 Months	12 Months		
Characteristics	Mean (SD)			p[1]	p[2]	Mean (SD)			p[1]	p[2]	Mean (SD)			p[1]	p[2]
**Age**				0.001	0.82				0.01	0.52				0.003	0.20
<50	62.3 (10.5)	56.4 (10.3)	54.6 (11.1)			55.4 (8.4)	53.1 (8.9)	53.8 (9.9)			59.1 (10.7)	53.0 (8.7)	52.1 (6.8)		
50-64	57.4 (8.6)	51.2 (9.9)	51.7 (9.4)			51.9 (7.9)	49.9 (9.5)	49.3 (10.3)			51.9 (10.3)	52.3 (10.3)	49.9 (10.1)		
≥65	54.1 (7.8)	49.6 (8.3)	49.5 (8.7)			48.8 (7.0)	48.9 (7.8)	48.1 (9.1)			48.9 (9.4)	49.6 (10.2)	47.1 (9.7)		
**Race**				0.75	0.43				0.97	0.13				0.48	0.99
White	56.6 (8.9)	51.0 (9.6)	51.3 (9.3)			51.0 (7.9)	49.7 (8.9)	49.4 (9.8)			51.4 (10.5)	51.2 (10.2)	49.1 (9.8)		
Non-White	56.7 (9.3)	53.1 (8.6)	47.8 (12.2)			51.1 (7.4)	53.2 (7.5)	47.0 (12.5)			54.0 (7.4)	53.5 (10.1)	46.9 (6.8)		
**Insurance status**				0.002	0.37				<0.001	0.04				<0.001	0.58
Medicare/Military/Tricare	54.5 (7.8)	50.0 (8.4)	49.9 (8.9)			49.1 (7.1)	49.3 (7.9)	48.5 (9.3)			49.3 (9.7)	49.9 (10.2)	47.4 (9.8)		
Medicaid/None/Self-pay	60.6 (10.6)	56.9 (11.8)	56.5 (10.5)			56.6 (9.8)	55.0 (11.5)	58.0 (11.8)			56.7 (11.3)	57.9 (10.7)	56.9 (8.7)		
Private	57.6 (8.9)	50.6 (9.4)	51.2 (9.3)			51.4 (7.3)	48.9 (8.4)	48.4 (9.4)			52.1 (10.2)	51.0 (9.5)	49.0 (9.4)		
**BMI**[1]				0.19	0.02				0.05	0.30				0.01	0.001
Normal weight	55.3 (10.9)	50.6 (8.2)	53.8 (6.4)			48.8 (7.0)	47.7 (7.9)	44.7 (6.2)			41.2 (7.4)	51.3 (12.4)	50.8 (12.1)		
Overweight	56.5 (9.4)	46.1 (8.0)	50.0 (8.1)			49.4 (6.9)	46.3 (5.7)	48.3 (7.5)			48.3 (7.9)	47.1 (8.9)	46.2 (9.2)		
Obese	56.8 (8.6)	52.4 (9.6)	51.3 (9.9)			51.6 (8.1)	50.9 (9.3)	50.0 (10.6)			53.1 (10.5)	52.4 (10.1)	49.7 (9.7)		
**Hypertension**				0.83	1.00				0.09	0.17				0.25	0.13
No	56.5 (9.0)	50.7 (9.6)	50.8 (9.0)			50.3 (7.8)	49.0 (8.0)	47.5 (9.5)			51.6 (10.3)	50.3 (9.8)	46.8 (9.5)		
Yes	56.7 (8.8)	51.4 (9.5)	51.5 (9.8)			51.6 (7.9)	50.5 (9.4)	51.0 (10.0)			51.4 (10.5)	52.1 (10.4)	51.1 (9.6)		
**Diabetes**				0.05	0.46				<0.001	0.27				0.00	0.56
No	56.3 (8.5)	50.3 (9.4)	50.7 (9.2)			50.2 (7.8)	48.9 (8.6)	48.1 (9.8)			50.4 (10.2)	50.6 (10.4)	48.2 (9.8)		
Yes	58.0 (9.9)	54.2 (9.4)	53.7 (9.8)			54.0 (7.4)	53.3 (8.8)	55.2 (7.8)			55.6 (10.2)	54.1 (8.9)	53.4 (8.3)		
**Alcohol use**				0.84	0.75				0.29	0.18				0.98	0.42
No	56.4 (8.9)	50.7 (9.9)	51.3 (9.6)			51.0 (8.3)	50.1 (9.3)	50.6 (10.5)			51.6 (11.1)	50.7 (10.7)	49.6 (10.3)		
Yes	57.0 (8.9)	51.5 (9.1)	50.9 (9.2)			51.0 (7.3)	49.3 (8.2)	47.4 (8.8)			51.4 (9.5)	52.0 (9.5)	48.2 (9.0)		
**Smoking status**				0.06	0.33				0.14	0.77				0.01	0.70
Never smoker	56.6 (8.4)	50.1 (9.3)	51.3 (9.1)			50.6 (7.5)	49.0 (8.2)	48.8 (9.0)			50.5 (10.0)	49.7 (9.8)	48.4 (9.1)		
Former smoker	54.6 (9.2)	51.8 (9.4)	50.2 (9.6)			51.4 (7.9)	50.7 (9.1)	50.4 (11.9)			52.1 (10.3)	52.6 (9.8)	49.5 (10.6)		
Current smoker	61.0 (9.6)	56.1 (10.1)	53.0 (11.9)			52.7 (9.9)	53.1 (11.3)	51.1 (13.4)			56.5 (11.6)	59.4 (9.8)	54.6 (13.6)		
**Distance from treatment facility**				0.35	0.81				0.18	0.48				0.74	0.72
<50 miles	57.6 (9.0)	51.2 (9.2)	51.7 (9.6)			51.8 (7.4)	50.3 (8.1)	49.6 (9.4)			51.8 (10.5)	51.9 (10.1)	47.8 (10.3)		
50-99 miles	55.9 (8.6)	51.4 (10.0)	51.3 (8.8)			50.9 (8.4)	49.8 (9.8)	49.9 (10.2)			51.8 (10.4)	51.0 (10.8)	50.8 (8.7)		
≥100 miles	55.1 (8.9)	49.0 (9.4)	47.4 (11.3)			47.9 (6.9)	47.9 (8.3)	43.9 (10.5)			48.9 (9.3)	49.6 (7.5)	47.1 (11.6)		
**Questionnaire type**				0.80	0.18				0.33	0.01				0.50	0.14
Computer-adaptive testing	57.3 (7.9)	51.1 (9.5)	50.1 (9.0)			51.4 (7.3)	49.9 (9.0)	47.3 (9.3)			52.0 (9.3)	50.8 (9.1)	47.5 (9.0)		
Short form	56.1 (9.6)	51.1 (9.6)	52.6 (9.7)			50.7 (8.3)	49.8 (8.7)	52.0 (10.1)			51.1 (11.2)	51.8 (11.1)	51.1 (10.4)		
**Histology**				0.92	0.43				0.69	0.11				0.66	<0.001
Low-grade endometrioid	56.5 (9.2)	51.0 (9.9)	51.5 (10.0)			50.9 (8.0)	49.6 (9.3)	49.5 (10.2)			51.6 (10.4)	50.1 (10.0)	49.4 (9.4)		
Other	57.2 (7.6)	51.6 (7.9)	50.0 (6.2)			51.3 (7.5)	50.6 (6.3)	48.4 (8.4)			51.2 (10.2)	56.3 (9.5)	47.6 (11.1)		
**Stage**				0.52	0.34				0.83	0.39				0.06	<0.001
I	56.9 (8.9)	50.9 (9.8)	51.5 (9.1)			51.1 (7.8)	49.7 (9.2)	49.6 (10.0)			51.4 (10.4)	49.9 (10.1)	48.9 (9.7)		
II/III/IV	55.8 (8.9)	51.7 (8.1)	49.5 (10.9)			50.6 (8.3)	50.4 (6.8)	47.5 (8.8)			52.0 (10.2)	57.6 (8.1)	49.6 (10.4)		
**Surgical approach**				0.11	0.73				0.19	0.16				0.17	0.76
Laparotomy	59.2 (7.8)	55.5 (7.1)	58.1 (8.7)			51.1 (9.1)	52.8 (6.3)	56.8 (9.6)			54.0 (8.5)	53.6 (10.6)	53.6 (15.2)		
Robotic-assisted laparoscopy	56.5 (8.9)	50.8 (9.6)	50.9 (9.3)			51.0 (7.8)	49.6 (8.9)	49.0 (9.8)			51.3 (10.5)	51.2 (10.2)	48.9 (9.5)		
**Lymph node staging**				0.02	0.59				0.11	0.19				0.01	0.02
Not performed	59.8 (11.0)	55.9 (9.0)	60.2 (11.6)			54.0 (9.0)	53.3 (10.0)	59.5 (7.9)			61.1 (10.1)	54.9 (11.0)	59.9 (10.7)		
Pelvic ± para-aortic lymphadenectomy	55.5 (9.1)	49.3 (8.7)	48.2 (8.6)			50.3 (7.4)	49.7 (8.0)	46.7 (9.4)			50.9 (9.4)	51.8 (10.4)	46.9 (9.1)		
Sentinel ± para-aortic lymphadenectomy	56.8 (8.4)	51.3 (9.8)	51.5 (9.1)			51.0 (7.9)	49.6 (9.0)	49.4 (9.8)			50.6 (10.3)	50.8 (10.0)	49.0 (9.5)		
**Chemotherapy**				0.91	0.34				0.90	0.32				0.23	<0.001
No	56.6 (8.8)	50.7 (10.1)	51.4 (9.5)			51.0 (7.9)	49.7 (9.7)	49.7 (10.2)			51.5 (10.2)	49.7 (10.0)	49.4 (9.2)		
Yes	56.8 (9.4)	52.2 (7.6)	50.3 (9.1)			51.1 (7.8)	50.5 (5.4)	48.0 (8.8)			51.7 (11.0)	56.3 (9.1)	47.8 (11.7)		
**Radiation**				0.17	0.78				0.19	0.06				0.60	0.001
No	57.2 (8.9)	51.3 (10.0)	51.6 (9.8)			51.3 (8.1)	49.9 (9.3)	50.2 (10.2)			51.7 (10.1)	50.5 (10.0)	50.0 (9.1)		
Yes	55.0 (8.8)	50.4 (8.2)	50.0 (8.1)			50.1 (7.0)	49.9 (7.3)	47.0 (8.5)			50.9 (11.3)	53.4 (10.5)	46.5 (11.0)		

	Sleep disturbance					Pain interference					Global pain				
	Baseline	6 Months	12 Months			Baseline	6 Months	12 Months			Baseline	6 Months	12 Months		
Characteristics	Mean (SD)			p[1]	p[2]	Mean (SD)			p[1]	p[2]	Mean (SD)			p[1]	p[2]
**Age**				<0.001	0.58				0.29	0.002				0.48	0.09
<50	57.9 (8.0)	54.1 (5.1)	54.3 (5.0)			57.0 (10.9)	48.0 (9.3)	47.9 (10.5)			4.6 (1.8)	1.5 (2.1)	1.5 (1.9)		
50-64	53.1 (9.2)	51.6 (9.6)	50.9 (9.2)			52.3 (10.5)	51.3 (10.1)	50.5 (9.8)			3.4 (2.8)	2.7 (2.7)	2.1 (2.3)		
≥65	48.4 (8.8)	48.0 (8.0)	45.9 (8.7)			49.5 (9.0)	50.9 (8.3)	49.4 (8.3)			2.6 (2.6)	2.3 (2.6)	2.2 (3.1)		
**Race**				0.34	0.20				0.30	0.17				0.39	0.53
White	51.5 (9.3)	50.3 (8.9)	49.4 (9.2)			51.4 (10.1)	50.7 (9.2)	50.0 (9.3)			3.1 (2.6)	2.4 (2.6)	2.2 (2.6)		
Non-White	57.9 (8.3)	53.3 (7.1)	47.3 (3.7)			58.9 (8.9)	53.2 (13.0)	43.5 (7.7)			5.2 (3.6)	4.0 (2.2)	0.0 (NA)		
**Insurance status**				<0.001	0.32				0.003	0.08				0.03	0.54
Medicare/Military/Tricare	48.8 (8.9)	48.2 (8.0)	46.1 (8.8)			49.8 (9.5)	51.2 (8.3)	49.5 (8.2)			2.6 (2.7)	2.3 (2.6)	2.2 (3.1)		
Medicaid/None/Self-pay	55.0 (10.9)	53.4 (11.6)	54.0 (7.2)			58.0 (11.1)	56.0 (10.3)	54.4 (11.7)			4.6 (2.6)	4.1 (2.8)	2.8 (1.9)		
Private	53.6 (8.7)	51.6 (8.4)	50.9 (9.0)			51.8 (9.9)	49.1 (9.5)	49.3 (9.6)			3.3 (2.5)	2.0 (2.4)	1.8 (2.3)		
**BMI**[1]				0.56	0.27				0.001	0.22				0.01	0.83
Normal weight	51.4 (8.7)	51.0 (6.8)	48.3 (10.0)			44.9 (8.5)	49.8 (8.1)	48.0 (5.8)			1.7 (2.0)	2.0 (2.5)	1.0 (1.2)		
Overweight	51.8 (7.5)	48.2 (10.4)	49.7 (6.7)			46.7 (8.3)	47.4 (8.3)	46.0 (8.5)			2.0 (1.9)	1.4 (1.8)	1.1 (2.1)		
Obese	51.8 (9.9)	50.9 (8.5)	49.3 (9.6)			53.5 (10.1)	51.7 (9.5)	51.0 (9.5)			3.7 (2.8)	2.8 (2.7)	2.6 (2.7)		
**Hypertension**				0.80	0.17				0.01	0.14				0.003	0.12
No	52.6 (9.4)	50.6 (7.9)	48.7 (9.4)			50.8 (10.1)	48.5 (8.4)	47.0 (8.0)			2.8 (2.3)	1.4 (1.9)	1.0 (1.2)		
Yes	51.1 (9.4)	50.3 (9.5)	49.9 (8.8)			52.5 (10.2)	52.7 (9.6)	52.5 (9.6)			3.5 (2.9)	3.2 (2.8)	3.2 (3.1)		
**Diabetes**				0.02	0.34				<0.001	0.78				0.004	0.45
No	51.1 (8.9)	49.5 (8.2)	49.0 (9.0)			50.3 (9.9)	49.4 (8.7)	48.6 (8.5)			2.9 (2.7)	2.0 (2.3)	1.9 (2.4)		
Yes	54.1 (10.8)	54.2 (10.3)	51.2 (9.5)			57.0 (9.5)	55.8 (9.9)	55.9 (10.7)			4.2 (2.2)	4.0 (3.1)	2.9 (3.0)		
**Alcohol use**				0.97	0.36				0.56	0.36				0.31	0.57
No	51.2 (10.2)	50.5 (9.6)	49.4 (9.7)			52.5 (11.0)	50.5 (10.0)	49.9 (9.5)			3.4 (3.0)	2.5 (2.7)	2.4 (2.8)		
Yes	52.4 (8.3)	50.3 (7.9)	49.0 (8.3)			50.7 (9.0)	51.2 (8.5)	50.0 (9.0)			3.0 (2.0)	2.4 (2.5)	1.7 (1.9)		
**Smoking status**				0.03	0.41				0.02	0.84				0.03	0.69
Never smoker	51.2 (8.8)	49.6 (8.4)	48.7 (9.2)			50.3 (9.4)	49.7 (8.5)	49.4 (8.9)			2.8 (2.4)	2.1 (2.2)	1.8 (2.3)		
Former smoker	50.6 (10.1)	50.6 (9.2)	50.3 (9.5)			53.7 (10.7)	52.1 (9.9)	49.2 (10.4)			4.2 (2.8)	3.3 (3.2)	3.3 (3.3)		
Current smoker	57.8 (9.6)	55.8 (9.6)	53.1 (7.0)			55.7 (12.1)	55.0 (11.8)	56.2 (8.6)			3.1 (3.5)	2.9 (3.0)	1.3 (2.3)		
**Distance from treatment facility**				0.73	0.50				0.94	0.52				0.69	0.51
<50 miles	52.4 (9.0)	50.0 (8.7)	49.5 (9.1)			51.2 (10.4)	51.1 (9.4)	50.1 (9.8)			3.2 (2.7)	2.4 (2.5)	1.9 (2.2)		
50-99 miles	51.5 (9.6)	51.3 (9.0)	49.6 (8.6)			52.7 (10.2)	50.7 (9.6)	49.3 (9.0)			3.3 (2.8)	2.8 (2.9)	2.4 (3.2)		
≥100 miles	50.0 (10.4)	48.9 (9.0)	46.8 (12.4)			50.0 (8.7)	49.9 (7.8)	50.9 (8.4)			2.9 (2.2)	1.3 (1.4)	2.0 (0.0)		
**Questionnaire type**				0.81	0.70				0.49	0.53					
Computer-adaptive testing	52.3 (9.5)	50.6 (10.0)	49.3 (8.5)			50.8 (10.2)	50.7 (9.3)	49.2 (9.6)							
Short form	51.4 (9.3)	50.3 (7.7)	49.4 (9.9)			52.5 (10.1)	50.9 (9.4)	50.6 (8.9)							
**Histology**				0.86	0.23				0.60	0.30				0.30	0.26
Low-grade endometrioid	51.5 (9.5)	50.4 (9.0)	49.1 (9.6)			51.6 (10.3)	50.3 (9.5)	49.6 (9.3)			3.2 (2.6)	2.2 (2.6)	1.8 (2.1)		
Other	52.8 (9.1)	50.7 (8.0)	50.2 (6.5)			52.1 (9.8)	52.9 (8.6)	50.6 (9.2)			3.3 (3.1)	3.4 (2.6)	3.5 (4.0)		
**Stage**				0.67	0.20				0.60	0.21				0.26	0.03
I	51.9 (9.3)	50.2 (9.2)	49.5 (9.4)			51.6 (10.6)	50.4 (9.4)	49.7 (9.3)			3.3 (2.8)	2.3 (2.6)	1.9 (2.6)		
II/III/IV	51.2 (9.7)	51.6 (7.0)	48.7 (7.7)			52.0 (8.7)	52.5 (8.7)	50.5 (9.6)			2.9 (2.4)	3.0 (2.7)	3.4 (2.6)		
**Surgical approach**				0.12	0.26				0.34	0.51				0.25	0.57
Laparotomy	57.8 (8.3)	53.6 (7.5)	51.3 (14.8)			55.5 (10.4)	53.9 (9.3)	49.9 (12.6)			3.8 (2.9)	3.6 (3.1)	2.7 (4.6)		
Robotic-assisted laparoscopy	51.3 (9.3)	50.2 (8.9)	49.3 (8.9)			51.4 (10.1)	50.6 (9.3)	49.8 (9.2)			3.2 (2.7)	2.4 (2.6)	2.1 (2.5)		
**Lymph node staging**				0.03	0.19				<0.001	0.37				0.00	0.39
Not performed	58.3 (11.1)	53.5 (8.8)	60.0 (5.7)			59.5 (9.4)	58.0 (11.0)	65.1 (5.8)			5.2 (2.5)	4.4 (2.9)	5.5 (3.0)		
Pelvic ± para-aortic lymphadenectomy	51.8 (8.9)	50.0 (7.4)	48.7 (8.7)			51.4 (9.1)	51.4 (8.6)	49.9 (10.1)			2.6 (2.6)	2.6 (2.7)	2.8 (4.4)		
Sentinel ± para-aortic lymphadenectomy	51.0 (9.1)	50.3 (9.4)	48.8 (9.0)			50.9 (10.4)	49.9 (9.2)	48.8 (8.4)			3.2 (2.6)	2.2 (2.5)	1.6 (1.8)		
**Chemotherapy**				0.64	0.09				0.26	0.18				0.55	0.36
No	51.6 (9.4)	50.2 (9.2)	49.6 (9.3)			51.1 (10.4)	49.9 (9.5)	49.7 (9.5)			3.2 (2.7)	2.2 (2.5)	2.0 (2.4)		
Yes	52.2 (8.8)	51.0 (7.4)	48.5 (8.5)			53.0 (9.5)	53.3 (8.6)	50.4 (8.7)			3.2 (2.6)	3.1 (2.8)	2.5 (3.1)		
**Radiation**				0.28	0.31				0.89	0.04				0.44	0.85
No	52.2 (9.0)	50.4 (8.8)	49.7 (8.6)			51.2 (10.4)	50.4 (9.5)	50.3 (9.5)			3.1 (2.5)	2.3 (2.6)	2.0 (2.3)		
Yes	50.2 (10.0)	50.2 (8.8)	48.4 (10.4)			52.9 (9.4)	52.0 (8.7)	48.5 (8.7)			3.7 (3.2)	2.9 (2.7)	2.6 (3.5)		

	Physical Function					Social Roles				
	Baseline	6 Months	12 Months			Baseline	6 Months	12 Months		
Characteristics	Mean (SD)			p[1]	p[2]	Mean (SD)			p[1]	p[2]
**Age**				0.82	0.43				0.83	0.25
<50	46.7 (8.3)	50.1 (8.4)	48.8 (9.3)			49.9 (8.1)	54.3 (10.3)	55.6 (10.2)		
50-64	47.6 (8.7)	47.2 (8.8)	48.1 (9.7)			53.1 (9.1)	52.6 (10.9)	54.8 (9.7)		
≥65	46.9 (8.2)	46.4 (8.5)	46.7 (7.3)			54.1 (8.3)	52.2 (8.9)	54.7 (9.1)		
**Race**				0.13	0.98				0.27	0.94
White	47.4 (8.3)	47.3 (8.5)	47.6 (8.9)			53.3 (8.5)	52.7 (9.8)	54.7 (9.5)		
Non-White	42.8 (10.7)	43.3 (11.5)	49.1 (1.7)			50.6 (13.6)	49.5 (16.9)	60.5 (1.8)		
**Insurance status**				0.002	0.74				0.01	0.04
Medicare/Military/Tricare	46.8 (8.2)	46.3 (8.6)	46.8 (7.2)			53.9 (8.6)	51.9 (9.1)	54.8 (8.9)		
Medicaid/None/Self-pay	43.4 (9.3)	42.8 (9.0)	41.6 (11.1)			50.5 (11.0)	46.7 (11.4)	49.5 (11.4)		
Private	48.6 (8.1)	49.1 (8.2)	49.3 (9.0)			53.2 (8.2)	54.8 (10.0)	55.8 (9.3)		
**BMI**[1]				<0.001	0.002				0.001	0.09
Normal weight	54.3 (7.0)	46.7 (11.4)	51.4 (10.7)			60.2 (5.8)	52.5 (12.9)	58.3 (7.6)		
Overweight	51.1 (6.9)	52.2 (6.6)	51.4 (6.0)			56.3 (6.4)	57.0 (8.2)	58.3 (7.6)		
Obese	45.7 (8.3)	45.9 (8.5)	46.3 (8.9)			51.8 (9.0)	51.6 (10.0)	53.6 (9.8)		
**Hypertension**				<0.001	0.12				0.01	0.23
No	49.5 (8.1)	49.9 (8.1)	51.6 (7.3)			54.2 (8.2)	54.4 (10.0)	57.4 (8.0)		
Yes	45.3 (8.2)	45.0 (8.5)	43.9 (8.4)			52.3 (9.1)	51.2 (10.0)	52.4 (10.1)		
**Diabetes**				<0.001	0.01				<0.001	0.45
No	48.2 (8.3)	48.2 (8.1)	49.1 (7.6)			54.2 (8.4)	53.8 (9.9)	56.2 (8.8)		
Yes	43.6 (7.8)	43.2 (9.8)	40.2 (10.3)			49.4 (8.9)	48.2 (9.8)	48.1 (9.7)		
**Alcohol use**				0.30	0.88				0.75	0.86
No	46.7 (8.8)	46.7 (8.8)	46.9 (8.7)			53.0 (9.4)	52.8 (10.4)	54.3 (10.3)		
Yes	47.8 (7.9)	47.6 (8.5)	48.4 (8.8)			53.4 (8.0)	52.5 (9.8)	55.7 (8.3)		
**Smoking status**				0.01	0.82				0.02	0.28
Never smoker	48.4 (8.4)	48.4 (8.0)	48.4 (8.2)			54.0 (8.0)	54.3 (9.6)	55.4 (9.1)		
Former smoker	45.0 (8.6)	45.3 (10.0)	47.3 (10.2)			51.5 (10.7)	50.0 (9.8)	54.9 (10.6)		
Current smoker	45.2 (7.1)	43.4 (8.1)	40.5 (7.7)			51.5 (8.1)	47.7 (11.5)	48.4 (7.8)		
**Distance from treatment facility**				0.90	0.39				0.44	0.73
<50 miles	47.5 (8.7)	46.3 (9.0)	47.6 (9.8)			52.4 (8.9)	52.4 (10.1)	54.8 (9.9)		
50-99 miles	46.9 (8.3)	48.2 (8.5)	47.8 (7.9)			53.6 (8.7)	52.6 (10.0)	53.9 (9.0)		
≥100 miles	47.6 (7.5)	47.3 (7.6)	47.1 (6.4)			54.7 (8.5)	53.8 (10.7)	59.8 (8.4)		
**Questionnaire type**				0.43	0.04				0.22	0.73
Computer-adaptive testing	45.9 (7.5)	46.8 (7.9)	48.0 (7.8)			53.6 (8.3)	53.5 (9.5)	55.7 (9.0)		
Short form	48.4 (9.0)	47.4 (9.4)	47.2 (9.9)			52.8 (9.2)	51.9 (10.6)	53.7 (10.0)		
**Histology**				0.48	0.004				0.04	0.04
Low-grade endometrioid	47.3 (8.4)	47.9 (8.8)	47.7 (8.9)			53.4 (8.8)	53.7 (9.9)	55.4 (9.6)		
Other	47.2 (8.6)	44.2 (7.6)	47.5 (8.2)			52.3 (8.6)	48.2 (9.6)	52.5 (8.5)		
**Stage**				0.01	<0.001				0.01	0.31
I	47.6 (8.6)	48.4 (8.6)	48.1 (9.0)			53.9 (8.8)	53.6 (10.3)	55.2 (9.6)		
II/III/IV	45.8 (7.8)	42.0 (6.8)	45.4 (7.0)			50.5 (8.0)	48.5 (8.0)	52.9 (8.2)		
**Surgical approach**				0.09	0.26				0.07	0.95
Laparotomy	44.5 (9.4)	42.1 (9.4)	45.7 (10.9)			49.5 (6.5)	48.7 (7.6)	50.5 (10.0)		
Robotic-assisted laparoscopy	47.4 (8.3)	47.5 (8.5)	47.7 (8.7)			53.5 (8.8)	52.9 (10.2)	55.0 (9.4)		
**Lymph node staging**				0.001	0.41				0.00	0.50
Not performed	41.2 (7.3)	39.8 (9.1)	33.0 (6.4)			44.9 (7.5)	47.4 (10.1)	41.1 (4.6)		
Pelvic ± para-aortic lymphadenectomy	47.9 (8.5)	46.7 (8.1)	47.3 (7.9)			53.2 (8.1)	52.6 (9.2)	54.8 (8.5)		
Sentinel ± para-aortic lymphadenectomy	47.7 (8.3)	48.0 (8.6)	48.7 (8.3)			54.1 (8.7)	53.1 (10.4)	55.8 (9.3)		
**Chemotherapy**				0.02	<0.001				0.02	0.11
No	47.7 (8.4)	48.7 (8.5)	47.9 (8.9)			54.0 (8.8)	54.0 (9.9)	55.1 (9.8)		
Yes	46.1 (7.9)	42.4 (7.6)	46.6 (8.1)			51.4 (7.8)	48.6 (9.6)	54.0 (8.1)		
**Radiation**				0.44	0.05				0.29	0.19
No	47.4 (8.5)	47.9 (9.0)	47.8 (9.0)			53.4 (8.7)	53.5 (10.3)	55.5 (9.3)		
Yes	47.0 (7.7)	45.1 (7.3)	47.3 (8.2)			53.2 (8.6)	50.3 (9.0)	53.2 (9.7)		

1 P-value for the main effect of the covariate, in a model including the covariate, time, and the interaction between the covariate and time

2 P-value for the interaction effect of the covariate and time, in a model including the covariate, time, and the interaction between the covariate and time
